# Green Synthesis of Biochar-Supported Nanoscale Zero-Valent Iron Using Tea Polyphenol for Efficient Cadmium Immobilization in Soil

**DOI:** 10.3390/nano15191460

**Published:** 2025-09-23

**Authors:** Ziyong Jia, Huizi Wang, Shupei Yuan, Weifeng Zhang, Daijun Zhang

**Affiliations:** 1 State Key Laboratory of Coal Mine Disaster Dynamics and Control, Chongqing University, Chongqing 400044, China; jiaziyong1121@163.com (Z.J.);; 2 Department of Environmental Science, College of Environment and Ecology, Chongqing University, Chongqing 400030, China

**Keywords:** biochar, nano-zero valent iron, Cd, remediation, tea polyphenols

## Abstract

With the increasing severity of cadmium (Cd) contamination in soil and its persistent toxicity, developing efficient remediation methods has become a critical necessity. In this study, sodium borohydride (NaBH_4_) and tea polyphenols (TP) were employed as reducing agents to synthesize biochar (BC)-supported nanoscale zero-valent iron (nZVI), denoted as BH_4_-nZVI/BC and TP-nZVI/BC, respectively. The effects of dosage, pH, and reaction time on Cd immobilization efficiency were systematically investigated. Both composites effectively stabilized Cd, significantly reducing its mobility and toxicity. Toxicity Characteristic Leaching Procedure (TCLP) results showed that Cd leaching concentrations decreased to 8.23 mg/L for BH_4_-nZVI/BC and 4.65 mg/L for TP-nZVI/BC, corresponding to performance improvements of 29.9% and 60.5%. The immobilization process was attributed to the reduction of Cd(II) into less toxic species, together with adsorption and complexation with oxygen-containing groups (-OH, -COOH, phenolic) on biochar. TP-nZVI/BC exhibited superior long-term stability, while maintaining slightly lower efficiency than BH_4_-nZVI/BC under certain conditions. Microbial community analysis revealed minimal ecological disturbance, and TP-nZVI/BC even promoted microbial diversity recovery. Mechanistic analyses further indicated that tea polyphenols formed a protective layer on nZVI, which inhibited particle agglomeration and oxidation, reduced the formation of iron oxides, preserved Fe^0^ activity, and enhanced microbial compatibility. In addition, the hydroxyl and phenolic groups of tea polyphenols contributed directly to Cd(II) complexation, reinforcing long-term immobilization. Therefore, TP-nZVI/BC is demonstrated to be an efficient, sustainable, and environmentally friendly amendment for Cd-contaminated soil remediation, combining effective immobilization with advantages in stability, ecological compatibility, and long-term effectiveness.

## 1. Introduction

Cadmium (Cd), a typical contaminant of heavy metals in soils, is particularly concerning due to its high mobility, bioavailability, and inability to self-stabilize [1]. Even at trace concentrations, Cd exhibits significant toxicity to crops, impairing their growth and entering the food chain, ultimately accumulating in humans [2,3]. This bioaccumulation poses severe health risks, including kidney damage, bone disorders, and carcinogenic effects [4]. Given its persistent environmental and biological threats, developing methods to immobilization of Cd in soil is critical for mitigating its impact [5,6].

Nanoscale zero-valent iron (nZVI) has been widely studied in environmental remediation, particularly for the treatment of heavy metal contamination in soils and water systems [7]. Its popularity arises from its unique physicochemical properties, including an extremely high surface area, strong reducing power, and high reactivity toward a wide range of contaminants [8,9]. In the context of heavy metal remediation, nZVI primarily functions through two mechanisms: chemical reduction and physical adsorption [10]. Its core–shell structure, consisting of a reactive metallic iron core and an iron oxide/hydroxide shell, allows it to interact with heavy metals such as cadmium (Cd), lead (Pb), chromium (Cr), and arsenic (As) [11,12]. Through reduction, nZVI can convert highly toxic metal ions, such as Cr(VI) and Cd(II), into their less toxic and less mobile elemental or lower oxidation state forms, effectively reducing their bioavailability [13]. In addition to reduction, nZVI exhibits strong adsorption properties due to its large specific surface area and porous structure, which allow for the sequestration of heavy metals onto its surface [14]. This dual functionality enables nZVI to address both immediate and long-term immobilization of heavy metals in contaminated soils [15].

Despite its efficacy, the practice of nZVI in the remediation of heavy metal-contaminated soils is hindered by several challenges [16]. One of the most significant limitations is its tendency to agglomerate due to magnetic interactions, which reduces its reactive surface area and overall effectiveness [17]. Furthermore, nZVI is prone to rapid oxidation when exposed to environment relevant conditions, which can decrease its reactivity over time [18]. To overcome these challenges, significant efforts have been made to modify nZVI to enhance its stability, dispersibility, and longevity. Among these strategies, the use of biochar (BC)-supported nZVI (nZVI/BC) has gained particular attention due to its environmental compatibility and synergistic properties [19,20]. BC derived from the pyrolysis of biomass under oxygen-limited conditions, serves as an excellent support matrix for nZVI [21,22]. Its high specific surface area, abundant oxygen-containing functional groups (e.g., hydroxyl, carboxyl, and phenolic groups), and intrinsic stability make it an ideal candidate for enhancing the performance of nZVI [23]. BC not only prevents the agglomeration of nZVI particles by providing physical separation but also increases the contact sites between nZVI and contaminants due to its rich porosity [24]. Furthermore, biochar contributes to mitigating the oxidative degradation of nZVI owing to its versatile redox capacity. In addition, biochar itself has inherent heavy metal immobilization capabilities, as it can adsorb and complex heavy metals through ion exchange, surface complexation, and precipitation mechanisms [25,26]. This dual functionality of nZVI/BC combining the reducing power of nZVI with the adsorption capacity of biochar creates a synergistic effect, allowing for more effective and sustainable remediation of heavy metal-contaminated soils [27].

nZVI can be synthesized through various methods, including chemical reduction, physical vapor deposition, microemulsion techniques, etc. [28]. Among these, chemical reduction using sodium borohydride (NaBH_4_) remains the most widely adopted method due to its simplicity, high efficiency, and ability to produce nZVI with a small particle size and high reactivity [29]. However, the NaBH_4_ based synthesis process is not without limitations. First, the high cost of NaBH_4_ and its limited availability make large-scale applications economically challenging. Second, the reaction often produces undesirable byproducts such as boron compounds, which may pose additional environmental risks. These drawbacks have spurred interest in alternative, more sustainable synthesis methods that can address these issues while maintaining the material’s high reactivity [30]. Tea polyphenols (TP), extracted from plant materials, have gained attention for their dual role as both a reducing agent and a stabilizer during nZVI synthesis [31]. The polyphenolic structure of TP provides abundant hydroxyl groups that can effectively reduce Fe(III)/Fe(II) to metallic iron, while the aromatic rings facilitate the capping and stabilization of the formed nZVI particles [32]. Compared to NaBH_4_, the use of TP offers several advantages, such as cost-effective, environmentally friendly, and eliminates the generation of hazardous byproducts. These features make TP an attractive alternative for synthesizing nZVI, particularly in applications requiring a sustainable and low-toxicity approach.

Accordingly, the performances of TP-nZVI/BC, a biochar-supported nanoscale zero-valent iron synthesized with tea polyphenols as a green reducing agent [33], were comprehensively evaluated for the remediation of Cd(II)-contaminated soils. Systematic investigations of dosage, pH, and reaction time confirmed its high immobilization efficiency, with toxicity leaching tests showing markedly lower Cd release compared with BH_4_-nZVI/BC. Mechanistic analyses further revealed that Cd stabilization was governed by reduction to less toxic species as well as adsorption and complexation with functional groups (–OH, –COOH, phenolic) on biochar, while the protective polyphenol layer enhanced the antioxidant capacity and inhibited Fe^0^ oxidation. In addition, microbial community analysis demonstrated that TP-nZVI/BC minimized ecological disturbance and even promoted microbial diversity recovery [34,35]. The novelty of this work lies in demonstrating that a natural reductant can not only substitute for NaBH_4_ in synthesizing nZVI/BC, but also impart advantages in stability and ecological compatibility. Overall, the findings provide new insights into the sustainable design of nZVI-based composites and underline the potential of TP-nZVI/BC as an environmentally friendly amendment for practical soil remediation applications.

## 2. Materials and Methods

### 2.1. Materials

Biomass charcoal(BC) was Biomass charcoal (BC) was purchased from Guangzhou Runfang Environmental Protection Technology Co., Ltd. (Guangzhou, Guangdong. China). Cadmium nitrate tetrahydrate (Cd(NO_3_)_2_·4H_2_O), cadmium chloride (CdCl_2_·2.5H_2_O), sodium hydroxide (NaOH), anhydrous calcium chloride (CaCl_2_), glacial acetic acid (CH_3_COOH), tea polyphenols (TP, C_7_H_8_N_4_O_2_), sodium borohydride (NaBH_4_), sodium acetate (CH_3_COONa), hydroxylamine hydrochloride (NH_2_OH·HCl), nitric acid (HNO_3_), hydrogen peroxide (H_2_O_2_), ammonium acetate (CH_3_COONH_4_), perchloric acid (HClO_4_), and hydrofluoric acid (HF) were all analytical-grade reagents obtained from Sinopharm Chemical Reagent Co., Ltd. (Shanghai, China) and used without further purification. TP was employed as a reference substance in FTIR analysis, while NaBH_4_ served as the conventional reductant to prepare BH_4_-nZVI/BC. CdCl_2_ and Cd(NO_3_)_2_ were used to prepare Cd-contaminated soils, and the remaining reagents were mainly applied for pH adjustment, extraction, and auxiliary analytical procedures (e.g., Tessia extraction).

### 2.2. Preparation of nZVI Loading Biochar by Sodium Borohydride

Biochar-supported nanoscale zero-valent iron was prepared using the sodium borohydride liquid-phase reduction method. Deionized water was pre-treated with nitrogen gas for 1 h to eliminate dissolved oxygen, and the sample preparation was carried out under anaerobic conditions. A total of 2.5 g of FeSO_4_·7H_2_O and 0.5 g of BC were added to 300 mL of deionized water, maintaining an iron-to-carbon ratio of 1:1. Iron ions were converted to nZVI by gradually adding 100 mL of NaBH_4_ solution. After reduction, the product was washed three times with deionized water and ethanol. The resulting material, biochar-supported nanoscale zero-valent iron (BH_4_-nZVI/BC) prepared by the sodium borohydride reduction method, was freeze-dried and stored in an anaerobic chamber.

### 2.3. Preparation of nZVI Loading Biochar by Tea Polyphenols

The preparation of TP involved brewing green tea leaves (from Wufeng, Yichang, Hubei Province, China) in deionized water at 80 °C for 30 min at solid-to-liquid ratio of 1:50 (*w*/*v*). The TP concentration in the resulting solution was determined using the Folin–Ciocalteu method (ISO Method 14502-1) and measured to be 2.62 ± 0.14 g/L. The filtration process was conducted in two steps: first, the solution was passed through a coffee filter to remove the tea leaves, and then it was filtered again using a sterile Millipore Durapore filter with a pore size of 0.22 μm. The filtered green tea solution was centrifuged at 3500 rpm for 10 min to bring it to room temperature. To prevent degradation of the tea polyphenols, a fresh green tea solution was prepared for each experiment. To synthesize biochar-supported nanoscale zero-valent iron (TP-nZVI/BC) using the tea polyphenol liquid-phase reduction method, 2.78 g of FeSO_4_·7H_2_O was added to 100 mL of an ethanol-water solution (volume ratio of ethanol to water: 3:7). Next, 0.56 g of polyvinylpyrrolidone (PVP) and 0.56 g of biochar were added to the solution, and the mixture was stirred to ensure homogeneous dispersion. Subsequently, a glass rod was used to stir the FeSO_4_·7H_2_O solution while 50 mL of the prepared tea polyphenol solution was rapidly added. The mixture was stirred thoroughly, and the desired nanoscale zero-valent iron particles were collected using a magnet. The particles were washed three times with deionized water, followed by three washes with anhydrous ethanol. The resulting production was freeze-dried.

### 2.4. Preparation of Soil Sample

The tested soil was collected from Chongqing City, China. First, the air-dried soil was manually cleaned to remove stones, grass roots, and other impurities. The soil was then ground with a mortar and passed through a 100-mesh (150 μm) sieve to obtain fine soil particles. A specific amount of sieved soil was accurately weighed into a beaker, and either a single or mixed heavy metal solution was added at a soil-to-solution ratio of 1 g:1 mL (mass:volume). The mixture was homogenized with a magnetic stirrer to ensure uniform distribution. After mixing, the samples were dried overnight in an oven (DHG-9070, Yi Heng, Shanghai, China). Once dried, the contaminated soils were retrieved and further ground to prepare them for subsequent solidification analysis. To obtain Cd(II)-contaminated soils with different concentrations, the soil was mixed with Cd(II) solutions of varying concentrations at a fixed solid-to-liquid ratio of 1:1 (g:mL) and homogenized with a magnetic stirrer. The mixture was then dried in an oven at 50 °C, and the dried Cd(II)-contaminated soils were ground to fine particles and stored in a dry environment for further use. This is because after the preparation of simulated contaminated soil and subsequent drying, some soil samples tended to form loose aggregates. Although such structures were unstable, it was still necessary to grind and sieve the dried soil prior to the experiments to ensure uniformity. The Cd concentrations in the prepared soils were 200 mg/kg, 400 mg/kg, and 600 mg/kg, respectively. For the preparation of Cd(II)-contaminated soils with different pH values, 2 mol/L HNO_3_ and 2 mol/L NaOH were used to adjust the pH of the Cd(II) solution to 4, 5, and 6. The pH-adjusted Cd(II) solutions were then mixed with a specific mass of soil at a solid-to-liquid ratio of 1:1 (g:mL), thoroughly homogenized, dried in an oven, and subsequently ground to fine particles for further use.

### 2.5. Soil Remediation and Analysis

Accurately weigh 1.0 g of contaminated soil into a 10 mL centrifuge tube and add the predetermined amount of solidification material. Subsequently, add 5.0 mL (5g) of ultrapure water to the centrifuge tube and mix thoroughly to ensure uniform blending of the soil, solidification material, and water. Place the centrifuge tube on a rotating shaker and stir at a speed of 30 ± 2 rpm at room temperature for a specific duration to facilitate the solidification reaction. For the remediation experiment, contaminated soil samples were mixed with BH_4_-nZVI/BC and TP-nZVI/BC and allowed to react for 24 h under the specified conditions. After the immobilization of Cd, the centrifuge tubes were removed and subjected to solid liquid separation by centrifugation at 5000 rpm. The supernatant was carefully collected for subsequent analysis, while the solid phase was dried and stored for further characterization. The supernatant is collected for subsequent analysis. The metal concentration in the supernatant is determined using flame atomic absorption spectroscopy (AAS) to evaluate the effectiveness of the solidification material in immobilizing metals in the soil.

For the toxicity leaching analysis of the optimal solidification material, the standard Toxicity Characteristic Leaching Procedure (TCLP) recommended by USEPA Method 1311 was employed to evaluate the leaching behavior of heavy metals in soil. Prior to the test, the pH of the soil sample was measured to determine the appropriate leaching agent. Specifically, 5.0 g of dried soil was mixed with 96.5 mL of deionized water, magnetically stirred for 5 min, and left to stand, after which the pH of the suspension was recorded. When the pH of the soil sample was below 5.0, Leaching Agent 1 was used; when the pH exceeded 5.0, Leaching Agent 2 was applied.

In the TCLP, two types of extraction fluids are defined to simulate different environmental conditions. Leaching Agent 1 consists of acetic acid partially neutralized with NaOH to pH 4.93. Its mechanism is to mimic acidic landfill environments where organic acids and weakly buffered conditions dominate. The presence of acetate ions promotes the solubilization of heavy metals through ligand complexation and pH-driven desorption from soil surfaces. Leaching Agent 2 is a more strongly acidic solution prepared by diluting acetic acid to pH 2.88 without neutralization. It represents harsher leaching conditions that may occur in environments with stronger acid inputs. The lower pH enhances proton exchange and disrupts metal–oxygen bonds in soil minerals, thereby increasing the release of metals from solid phases.

The leaching agents were prepared as follows: Leaching Agent 1 was obtained by diluting 5.7 mL of glacial acetic acid and 64.3 mL of 1 M NaOH to a final volume of 1 L, with the pH adjusted to 4.93 ± 0.05 using 1 M NaOH or HNO_3_. Leaching Agent 2 was prepared by diluting 5.7 mL of glacial acetic acid to 1 L, with the final pH maintained at 2.88 ± 0.05. In practice, Leaching Agent 2 is most commonly used, as soil pH values are generally higher than 5.0. For the leaching test, 0.5 g of dried soil was combined with 10 mL of deionized water (solid–liquid ratio of 20:1, L/kg) in a polyethylene (PE) tube. The tubes were mounted on a rotary shaker and agitated at 30 ± 2 rpm at room temperature for 16 ± 2 h. Following extraction, the suspensions were centrifuged and filtered, and the concentrations of heavy metals in the supernatants were determined by AAS. The experimental process is illustrated in Figure S1.

### 2.6. Characterization and Morphology

The materials before and after the reaction using for immobilization of Cd in the soil were characterized by X-ray diffraction (XRD, D8 Advance, Bruker, Germany) using Cu Kα radiation to analyze the change in the crystalline phase. Scanning electron microscopy (SEM, JSM-5510LV, JEOL, Akishima, Japan) and energy dispersive spectrometry (EDS, Pottsville, PA, USA) were employed to examine the surface morphology of the material both prior to and following the reaction. Functional groups were recorded using a Fourier transform infrared spectrometer (FT/IR-670Plus, Tokyo, Japan) in the range of 400–4000 cm^−1^. The bet sureface area was measured by Micromeritics ASAP 2460 (Norcross, GA, USA). In addition, the variations in the electronic binding energy of iron and oxygen elements were analyzed using an X-ray photoelectron spectrometer (XPS) (Thermo Scientific K-Alpha, Waltham, MA, USA).

## 3. Results and Discussion

### 3.1. Characterizations

**Figure 1:**
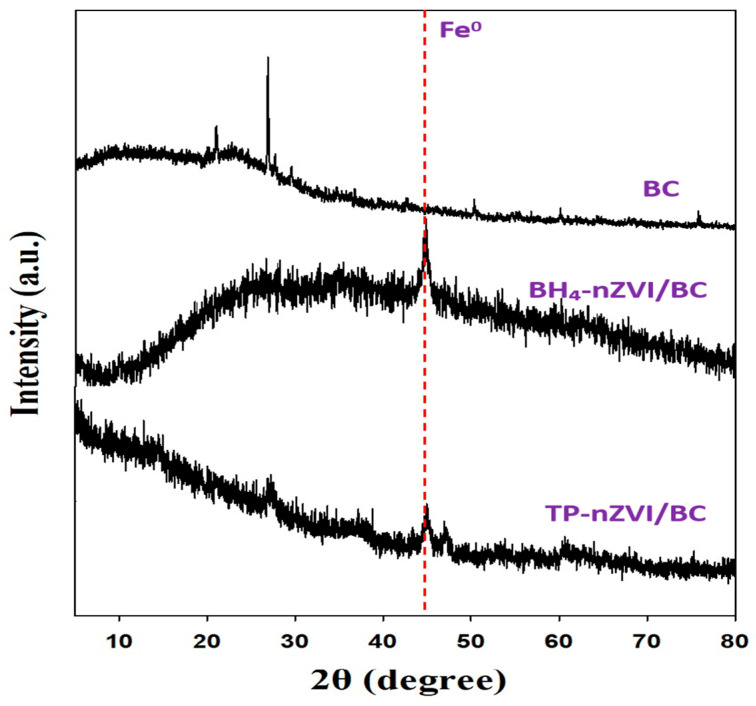
The XRD patterns of BC, BH_4_-nZVI/BC and TP-nZVI/BC.

The XRD analysis showed that the biochar exhibited a distinct diffraction peak at 26.6°, attributed to the (002) crystal plane of graphitic carbon (Figure 1). After loading nanoscale zero-valent iron, BH_4_-nZVI/BC displayed a characteristic diffraction peak of nZVI at 44.9° [36]. The broad width of this peak indicates that nZVI particles possess an amorphous structure, consistent with reports that nZVI typically exhibits an amorphous state or low crystallinity [37]. Similarly, in TP-nZVI/BC, the characteristic diffraction peak of nZVI was also observed, with a similarly broad peak width.

**Figure 2:**
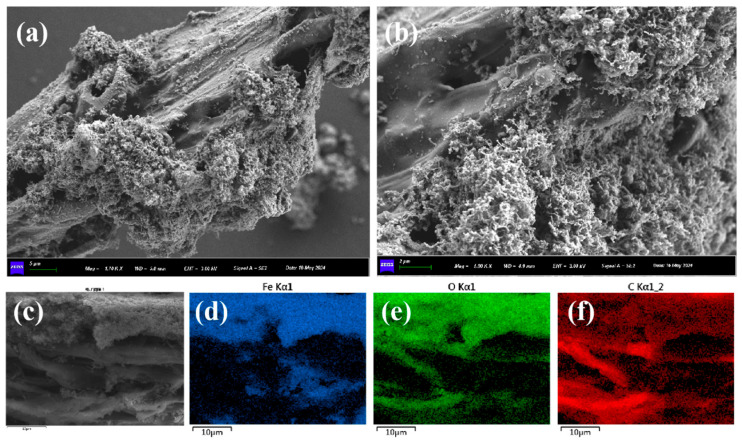
(**a**–**c**) are SEM images of BH_4_-nZVI/BC; (**d**) Fe, (**e**) O, (**f**) C elemental EDS mapping images.

The morphology of BH_4_-nZVI/BC was observed under SEM at different magnifications (Figure 2). A large number of iron nanoparticles were found to be relatively uniformly dispersed on the surface of the biochar, which exhibited a tubular structure with numerous microvoids on its surface. The rough texture and natural porosity of the biochar provided abundant attachment sites for the iron nanoparticles. EDS mapping revealed that the distribution of Fe elements corresponded to the distribution of the nanoparticles, and most of the C elements were located near Fe, further confirming that zero-valent iron was successfully loaded onto the biochar surface. Moreover, the nZVI on the BH_4_-nZVI/BC surface was present in spherical or chain-like structures, uniformly distributed across the biochar surface. This uniform dispersion reduced nanoparticle agglomeration and highlighted the role of biochar as a supporting matrix [38].

**Figure 3:**
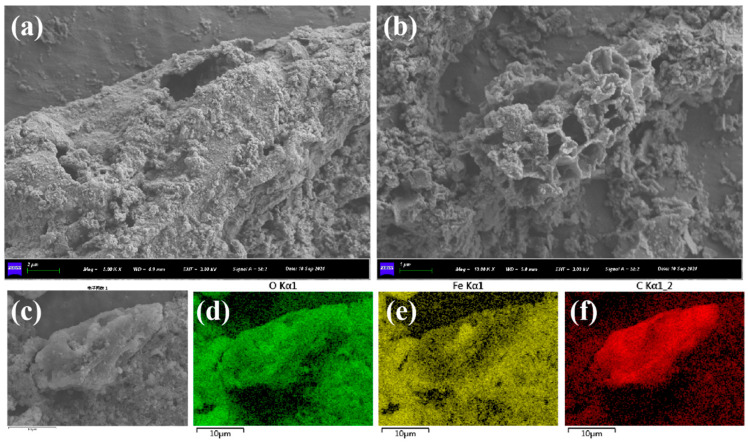
(**a**–**c**) are SEM images of TP-nZVI/BC; (**d**) Fe, (**e**) O, (**f**) C elemental EDS mapping images.

The SEM images in Figure 3 reveal the microstructure of the TP-nZVI/BC surface. The surface appears rough with distinct textures and wrinkles, likely resulting from structural changes in the biochar during the high-temperature pyrolysis process. The observations indicate a close integration between the nZVI and BC, showcasing the porous structure of biochar while revealing the uniform distribution of nZVI particles, forming a unique composite morphology. This structural feature demonstrates that nZVI can be evenly loaded onto the biochar, thereby enhancing the adsorption properties of the material. Additionally, EDS mapping (Figure 3d–f) of the material before reaction shows that Fe elements are uniformly distributed, indicating strong composite stability between nZVI and biochar, with tight bonding between Fe and C elements. This uniform distribution of Fe likely enhances the immobilization efficiency of the material by providing more active sites for the stabilization of heavy metals in soil.

**Figure 4:**
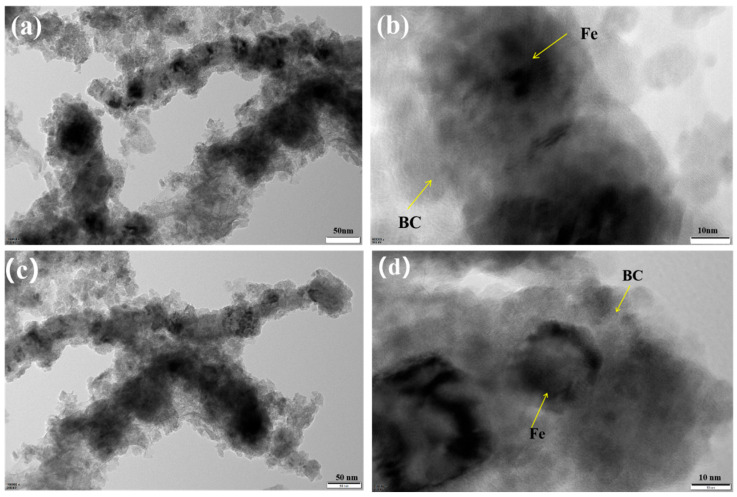
TEM images of BH_4_-nZVI/BC (**a**,**b**) and TP-nZVI/BC (**c**,**d**).

**Figure 5:**
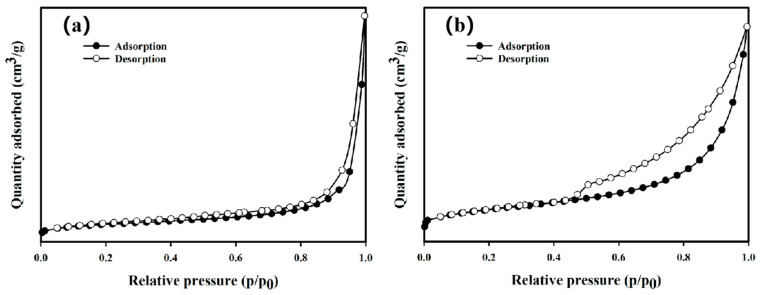
BET of (**a**) BH_4_-nZVI/BC and (**b**) TP-nZVI/BC.

As shown in Figure 4, the particle size of nZVI in both BH_4_-nZVI/BC and TP-nZVI/BC ranges from 50 nm to 100 nm. Additionally, it can be observed that the nanoscale zero-valent iron is well-distributed within the biochar matrix, consistent with previous literature reports [39]. As shown in Figure 5a, the specific surface area of BH_4_-nZVI/BC is 51.17 m^2^/g, indicating a relatively high external and internal porous surface area, which provides significant advantages for adsorption and related applications. The material’s total pore volume is 0.290 cm^3^/g, and the average pore size is 22.50 nm, classifying it as a mesoporous structure (pore size between 2 and 50 nm) [40]. This mesoporous structure offers numerous active sites, enhancing the material’s adsorption capacity and reaction efficiency. In comparison, the specific surface area of TP-nZVI/BC is 88.26 m^2^/g, which is higher than that of BH_4_-nZVI/BC, with a total pore volume of 0.266 cm^3^/g and an average pore size of 11.58 nm, also within the mesoporous range (Figure 5b). These results indicate that both BH_4_-nZVI/BC and TP-nZVI/BC possess suitable pore sizes to facilitate the adsorption process efficiently.

### 3.2. Immobilization of Cd by BH_4_-nZVI/BC and TP-nZVI/BC and in the Soil

**Figure 6:**
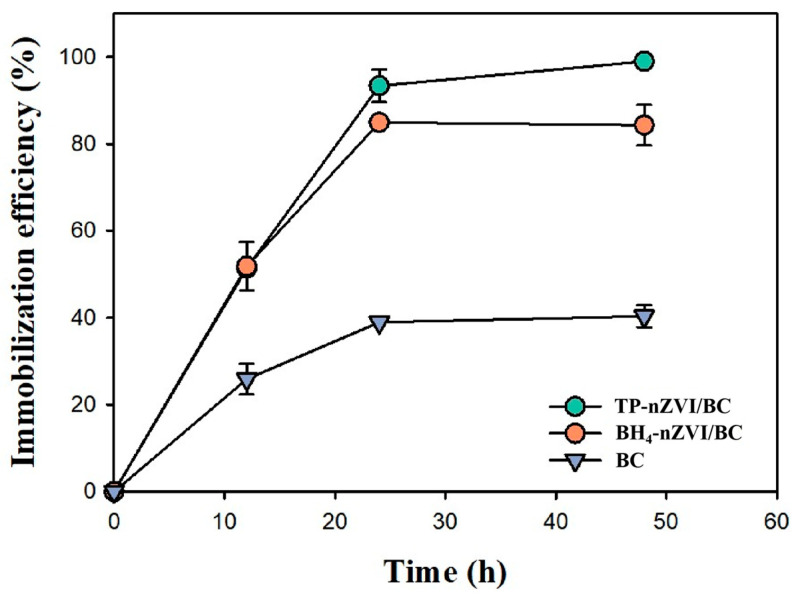
Immobilization efficiency of BH_4_-nZVI/BC, TP-nZVI/BC and BC for Cd containing soils with different Cd concentrations (Dosage 0.07 g, Initial concentration of Cd(II)-contaminated soil: 400 mg/kg).

Figure 6 illustrates the variation in Cd immobilization efficiency of BH_4_-nZVI/BC, TP-nZVI/BC, and BC over time. Among the materials, BH_4_-nZVI/BC demonstrated the highest immobilization efficiency, reaching nearly 100% at 30 h and maintaining stability up to 48 h. The immobilization efficiency increased rapidly within the first 10 h, indicating strong initial reactivity. In comparison, TP-nZVI/BC achieved a maximum immobilization efficiency of approximately 84.3%, stabilizing between 20 and 30 h. This slightly lower efficiency may be attributed to a reduced nZVI content or slower reaction kinetics. BC exhibited the poorest performance, with a gradual increase in efficiency over time, stabilizing at 40.3%, primarily due to physical adsorption and complexation. The superior performance of BH_4_-nZVI/BC is attributed to the synergistic effects of nZVI’s strong reductive capacity and biochar’s high adsorption capacity. nZVI rapidly converts Cd(II) into stable forms, while biochar further enhances Cd immobilization through surface functional groups.

### 3.3. Effect of the Initial Concentration of Cd in Soil on the Remediation

**Figure 7:**
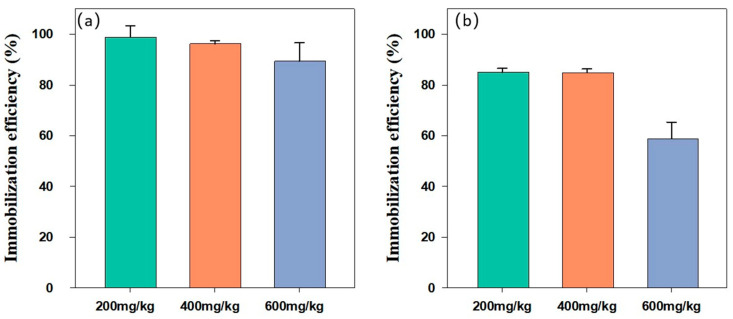
Immobilization efficiency of (**a**) BH_4_-nZVI/BC and (**b**) TP-nZVI/BC for Cd containing soils with different Cd concentrations (Dosage 0.07 g, reaction time 24 h).

Figure 7 presents the Cd immobilization efficiency of BH_4_-nZVI/BC (a) and TP-nZVI/BC (b) in soils contaminated with different Cd concentrations (200 mg/kg, 400 mg/kg, and 600 mg/kg). For BH_4_-nZVI/BC, the immobilization efficiency remained above 90% across all Cd concentrations, with only a slight decline as Cd concentration increased. This performance can be attributed to the high reductive capacity and stability of BH_4_-nZVI/BC. Its ability effectively transforms Cd(II) into more stable forms with reduced toxicity or mobility, potentially involving co-precipitation with iron oxides or adsorption complexes, and other indirect reduction products. Additionally, the biochar’s high specific surface area and abundant surface functional groups (e.g., hydroxyl and carboxyl groups) further enhance Cd adsorption, synergistically improving immobilization efficiency [41]. In contrast, the immobilization efficiency of TP-nZVI/BC decreased significantly with increasing Cd concentrations, dropping from approximately 85% at 200 mg/kg to about 60% at 600 mg/kg. This decline may be attributed to the initial stability and active sites provided by TP-nZVI/BC, which effectively immobilize Cd in low-concentration environments. However, in high-Cd concentration scenarios, the active sites on the material surface may gradually become saturated with Cd, limiting further adsorption and reduction processes, thus reducing the immobilization efficiency [42].

### 3.4. Effect of the Initial pH of Cd in Soil on the Remediation

**Figure 8:**
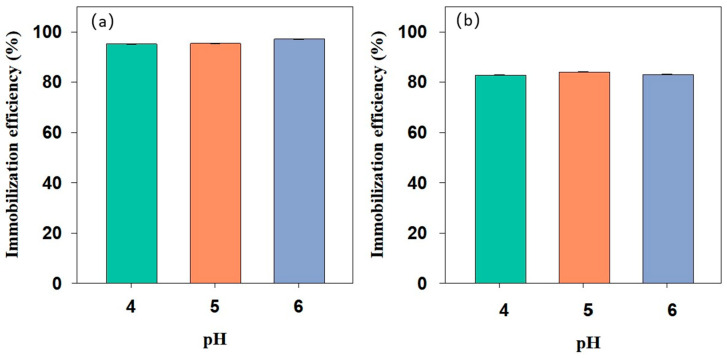
Influence of the initial pH on the immobilization efficiency of Cd by (**a**) BH_4_-nZVI/BC and (**b**) TP-nZVI/BC.

pH is a key environmental parameter determining the speciation of heavy metals and significantly influencing the electrochemical properties of passivating agents. Figure 8 illustrates the effect of initial pH (4, 5, and 6) on the Cd immobilization efficiency of BH_4_-nZVI/BC and TP-nZVI/BC. As shown in the figure, both materials exhibited highly efficient and stable Cd immobilization within the tested weakly acidic pH range, with immobilization efficiencies approaching 100% and minimal fluctuation with changes in pH. This indicates that both BH_4_-nZVI/BC and TP-nZVI/BC have strong adaptability and remarkable stability for Cd immobilization under varying environmental conditions.

### 3.5. Effect of Dosage on the Immobilization

**Figure 9:**
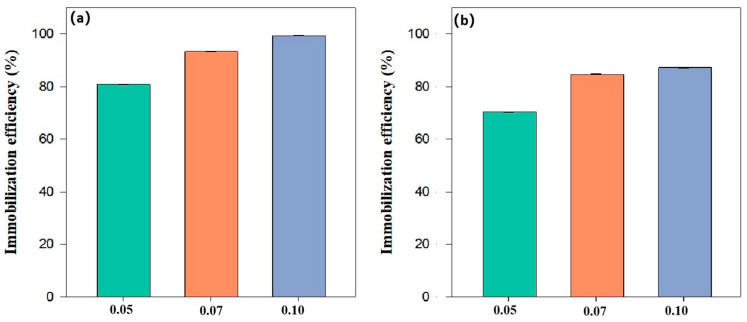
Influence of the dosage on the remediation performance of Cd by (**a**) BH_4_-nZVI/BC and (**b**) TP-nZVI/BC (Initial concentration of Cd(II)-contaminated soil: 400 mg/kg, reaction time: 24 h).

Figure 9 illustrates the Cd immobilization efficiency of BH_4_-nZVI/BC (a) and TP-nZVI/BC (b) at different dosages (0.05 g, 0.07 g, and 0.10 g in 1 g soil). The results show that the Cd immobilization efficiency of both materials increased significantly with higher dosages. For BH_4_-nZVI/BC, the efficiency was approximately 85% at 0.05 g and increased to nearly 100% at 0.10 g, indicating that a higher dosage provides more nZVI particles and biochar adsorption sites, significantly enhancing the Cd immobilization capacity. Similarly, for TP-nZVI/BC, the efficiency improved from around 70% at 0.05 g to approximately 90% at 0.10 g. Although the immobilization efficiency of TP-nZVI/BC was slightly lower than that of BH_4_-nZVI/BC, increasing the dosage effectively enhanced its activity and adsorption capacity [43]. This trend can be attributed to the higher material dosage providing more active components, such as nZVI and biochar, thereby offering additional reaction sites and adsorption surface area. The immobilization mechanism involves nZVI reducing Cd(II) to less toxic and less mobile metallic forms, while biochar enhances Cd immobilization through surface complexation and electrostatic adsorption. The slightly lower efficiency of TP-nZVI/BC compared to BH_4_-nZVI/BC may be due to a lower quantity of active components in the material.

### 3.6. The Result of TCLP Leaching

**Figure 10:**
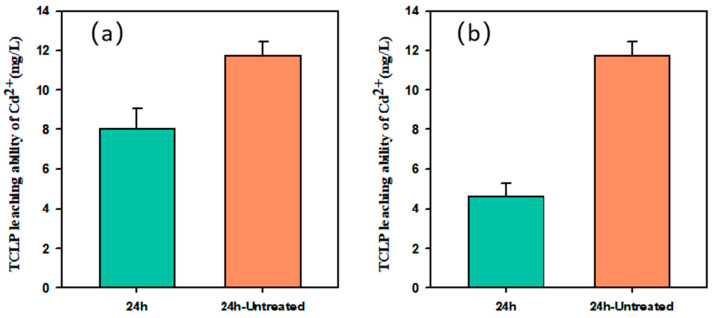
TCLP leachability of Cd before and after the soil samples were treated, (**a**) BH_4_-nZVI/BC and (**b**) TP-nZVI/BC (Initial concentration of Cd(II)-contaminated soil: 400 mg/kg, reaction time: 24 h.

The TCLP is a widely used method for assessing the effectiveness of toxic metal immobilization in contaminated soils [44]. As shown in Figure 10, the Cd(II) leaching concentration in untreated soil reached 11.75 mg/L. After 24 h of treatment, the concentration decreased to 8.23 mg/L with BH_4_-nZVI/BC and 4.65 mg/L with TP-nZVI/BC, corresponding to performance improvements of 29.9% and 60.5%, respectively. These results indicate that both composites effectively reduced Cd mobility, while TP-nZVI/BC exhibited markedly superior stabilization. Similar observations have been reported in other studies. For example, CaFe-LDH reduced TCLP-extractable Cd from 142.30 to 48.13 mg/L [45]. Xu et al. found that the initial TCLP-leachable Cd in soil (0.08 mg/L) decreased to 0.059, 0.063, 0.051, 0.052, and 0.035 mg/L after treatment with nZVI, biochar, nZVI/BC, sulfidized nZVI, and S-nZVI/BC, corresponding to reductions of 25.32%, 20.25%, 35.44%, and 34.18%, respectively [46]. Similarly, Shao et al. reported that in municipal sludge, Cd leaching decreased from 0.68 to 0.62 mg/kg after nZVI/BC treatment [47].

To better understand these improvements, it is necessary to consider the underlying immobilization mechanisms. During the remediation process, biochar and iron materials in the soil form stable complexes with Cd (Biochar–COOCd^+^, FeOCd^+^ and Cd(OH)_2_), thereby reducing its mobility and leaching toxicity [48]. The superior performance of TP-nZVI/BC compared with BH_4_-nZVI/BC can be attributed to the protective polyphenol layer formed during synthesis, which enhances the antioxidant capacity and dissolution resistance of nZVI. This stabilization effect suppresses Fe^0^ oxidation and passivation, thereby strengthening long-term Cd immobilization.

### 3.7. Heavy Metal Speciation in Soil

**Figure 11:**
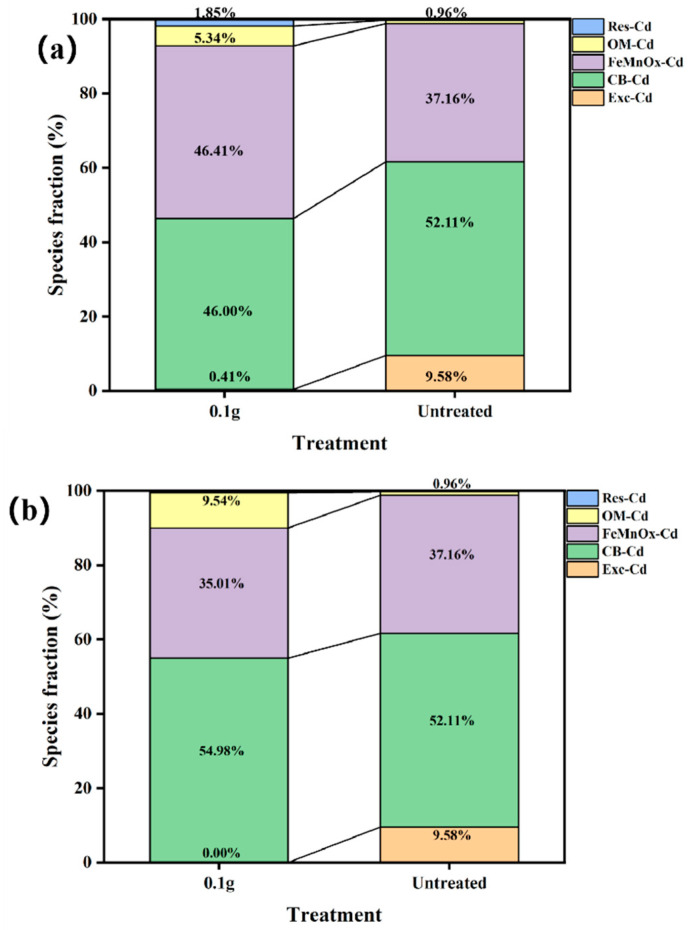
Percentage of different forms of Cd in the soil before and after treatment (**a**) BH_4_-nZVI/BC and (**b**) TP-nZVI/BC.

The biological toxicity of heavy metals in soil is not only related to their TCLP leaching but also directly linked to their speciation distribution, which determines their environmental impact [49]. The speciation of Cd(II) in soil was analyzed using the Tessier sequential extraction method, categorizing heavy metals into exchangeable (Exc), carbonate-bound (CB), Fe-Mn oxide-bound (FeMnOx), organic-bound (OM), and residual (Res) forms [50]. As shown in Figure 11, the application of biochar-supported nanoscale zero-valent iron (BH_4_-nZVI/BC) significantly influenced the speciation distribution of Cd in the soil. The results revealed that with an increasing dosage of the stabilizer, the percentage of exchangeable Cd decreased significantly from 10% to 0%, indicating a substantial reduction in the bioavailability of Cd in the soil. In untreated soil, the residual Cd content was 0%, which increased to 2% after the application of BH_4_-nZVI/BC, suggesting that the stabilizer promoted the transformation of Cd into a more immobile and less bioavailable residual form. Furthermore, the organic-bound Cd content increased from 0% to 5% after treatment, likely indicating enhanced binding of Cd with soil organic matter, further reducing its bioavailability. The carbonate-bound Cd content showed minimal change, suggesting that in the tested pH range, carbonate-bound Cd was relatively stable. Notably, the Fe-Mn oxide-bound Cd content increased significantly, indicating that the added materials interacted with Cd in the soil, potentially through adsorption and co-precipitation mechanisms, enhancing the transformation of Cd into Fe-Mn oxide-bound forms. Similarly, the addition of TP-nZVI/BC resulted in significant changes in the speciation distribution of Cd. Specifically, the exchangeable Cd content decreased from 9.6% to 0.0%, the carbonate-bound Cd content decreased from 52.1% to 49.6%, while the Fe-Mn oxide-bound Cd content increased from 37.2% to 38.5%. The organic-bound Cd content increased notably from 0.1% to 11.4%, whereas the residual Cd content remained relatively low. These results indicate that, under the stabilizing effect of the passivators, Cd primarily transformed from exchangeable and carbonate-bound forms into more stable Fe-Mn oxide-bound and organic-bound forms, significantly reducing its environmental mobility and migration risk [51].

### 3.8. Soil Microbial Community After Treatment

**Figure 12:**
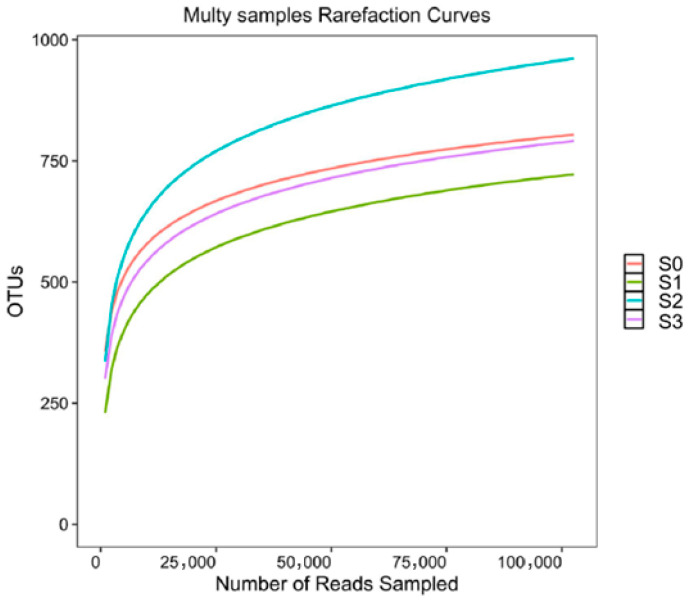
Rarefaction curves of different soil samples (S0, S1, S2, and S3 represent clean soil, Cd(II)-contaminated soil, TP-nZVI/BC-treated Cd(II)-contaminated soil, and BH_4_-nZVI/BC-treated Cd(II)-contaminated soil, respectively).

Soil harbors a diverse range of microorganisms that play a crucial role in maintaining ecosystem balance and promoting soil health. The sensitivity of soil microbial communities to external factors or human disturbances can serve as an effective indicator of soil ecological risk and health. In this study, 16S rRNA gene sequencing was performed using the Illumina MiSeq platform to analyze microbial diversity and assess the impact of BH_4_-nZVI/BC and TP-nZVI/BC treatments on soil microbial communities. Figure 12 shows the relationship between the Alpha diversity index and gene sequence numbers, where the rarefaction curves stabilize as sequencing depth increases, indicating that the sequencing depth was sufficient and that microbial species had reached saturation, effectively reflecting changes in soil microbial community structure. Heavy metal contamination is a significant factor influencing soil microbial communities, often manifested in changes in microbial community richness and diversity. Table 1 lists the microbial diversity indices for all soil samples. Good’s coverage index for all samples was above 0.99, indicating no significant differences in microbial coverage across samples, which reflects the high coverage of species detected in this sequencing result [52]. In terms of microbial community richness, the Chao1 and ACE indices of S2 were lower than those of S0 and S1, but compared to S3, S2 exhibited higher microbial diversity. This suggests that while the microbial richness in S2 declined after Cd(II) contamination, the restoration of soil-adapted communities due to TP-nZVI/BC treatment led to increased microbial community stability and enhanced resistance to pollution [53]. This indicates that the tea polyphenol modification in TP-nZVI/BC plays a significant role in influencing the microbial community. Tea polyphenols, as natural antioxidants and chemical modifiers, can enhance the surface properties of zero-valent iron, improving its capacity for heavy metal adsorption and reduction, while also reducing oxidative damage to microorganisms caused by iron oxides. The modification by tea polyphenols not only improved the remediation efficacy of BH_4_-nZVI/BC but also facilitated the restoration and maintenance of microbial diversity. Therefore, S2 showed better microbial community recovery and heavy metal degradation capabilities during the remediation process, with significant ecological restoration advantages. In contrast, the microbial community diversity of S3, treated with BH_4_-nZVI/BC, was significantly reduced, likely due to the transformation of BH_4_-nZVI/BC into iron oxides (such as FeOOH and Fe_2_O_3_) and the accumulation of iron and iron oxides, leading to oxidative damage to microbial cells, which suppressed microbial growth and reduced diversity [54]. Thus, S2 exhibited greater potential for remediation, particularly in the recovery and stability of microbial communities, with tea polyphenol modification playing a key positive role in this process.

**Figure 13:**
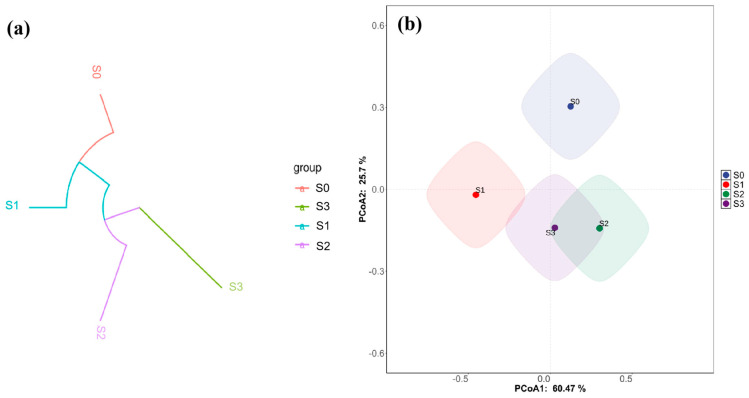
Beta diversity analysis under different treatments (**a**) Multi-sample clustering tree based on Bray–Curtis distance, (**b**) PCoA analysis plot (S0, S1, S2, and S3 represent clean soil, Cd(II)-contaminated soil, TP-nZVI/BC-treated Cd(II)-contaminated soil, and BH_4_-nZVI/BC-treated Cd(II)-contaminated soil, respectively).

To further investigate the similarities and differences in microbial community structure at the OTU level, Beta diversity analysis was performed. Hierarchical clustering analysis based on the Beta diversity distance matrix was conducted using a dendrogram Figure 13a [55]. It can be observed that the different treatment groups show a clear separation. Based on Beta analysis, the PCoA analysis results, drawn using R software, are shown in Figure 13b. In the PCoA analysis of soil microbial communities, the closer the sample points are, the more similar their community structures. In the TP-nZVI/BC-treated group, the first principal component (PCoA1) explained 60.47% of the variation in sample differences, and the second principal component (PCoA2) contributed 25.7%. From the figure, it is evident that S0 is distanced from the other three groups, indicating that the microbial community in the clean soil (S0) differs significantly from those in the other treated and polluted soils. The four soil types exhibit distinct microbial structures, which is consistent with the clustering tree results. Thus, a further analysis was conducted to explore the changes in the microbial community under different treatments. Figure 13 shows the Beta diversity analysis results of soil microbial communities under different treatments, including the multi-sample clustering tree based on the Bray–Curtis distance matrix (Figure 13a) and the principal coordinate analysis (PCoA) plot (Figure 13b). This analysis was conducted to examine the similarity and differences in microbial community structures between soil samples from different treatment groups. In Figure 2a, the multi-sample clustering tree based on the Bray–Curtis distance matrix displays the clustering of soil samples. From the clustering tree, it is apparent that all samples exhibit a distinct separation, indicating significant differences in the microbial community structures between the treatment groups. Specifically, the S0 (clean soil) sample is more distant from the other three polluted soil samples (S1, S2, S3), suggesting that the microbial community in unpolluted soil differs greatly from that in polluted soils. Figure 13b shows the PCoA plot based on the analysis. In the PCoA analysis, the first principal component (PCoA1) explains 60.47% of the variation, and the second principal component (PCoA2) contributes 25.7%. It can be seen that the S0 sample point is farther from the other three groups, indicating that clean soil and the Cd(Ⅱ)-polluted soil, along with its subsequent treatments, have distinct microbial community structures. The S1 (Cd(Ⅱ)-polluted soil), S2 (TP-nZVI/BC-treated Cd(Ⅱ) soil), and S3 (BH_4_-nZVI/BC-treated Cd(Ⅱ) soil) samples cluster together, suggesting that the microbial communities in these treated soils exhibit similar structural changes, even though their community structures still differ. S2 and S3 samples are closer to each other in the PCoA plot, indicating that the microbial communities in TP-nZVI/BC and BH_4_-nZVI/BC-treated soils tend to be more similar in certain aspects, especially in the heavy metal remediation process. The tea polyphenol-modified BH_4_-nZVI/BC and unmodified BH_4_-nZVI/BC share some common characteristics. Overall, the analysis in Figure 13 indicates that heavy metal contamination and different treatment methods significantly impact the structure of soil microbial communities. Although TP-nZVI/BC (S2) and BH_4_-nZVI/BC (S3) exhibit similarities in community structure, the TP-nZVI/BC-treated sample (S2) shows relatively better community recovery, while the differences between Cd(Ⅱ)-polluted soil (S1) and clean soil (S0) are more pronounced. This suggests that TP-nZVI/BC treatment can restore soil microbial community stability to some extent, mitigating the negative impact of heavy metal contamination on microbial communities.

**Figure 14:**
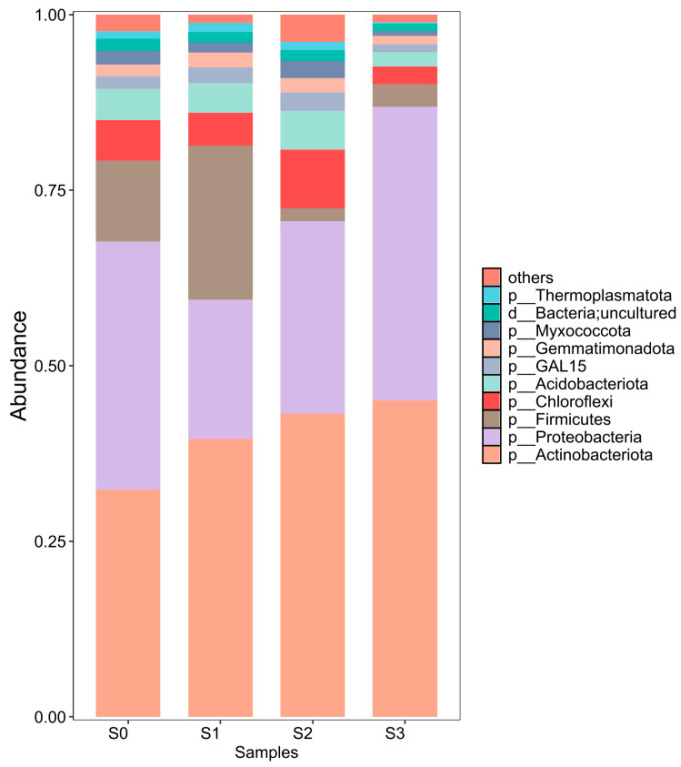
Relative abundance of bacterial phyla in soil at the phylum level (S0, S1, S2, and S3 represent clean soil, Cd(II)-contaminated soil, BH_4_-nZVI/BC-treated Cd(II) soil, and TP-nZVI/BC-treated Cd(II) soil, respectively).

Figure 14 shows the relative abundance of bacterial phyla in soil under different treatments. The figure illustrates the bacterial community composition in clean soil (S0), Cd(Ⅱ)-contaminated soil (S1), TP-nZVI/BC-treated Cd(Ⅱ) soil (S2), and BH_4_-nZVI/BC-treated Cd(Ⅱ) soil (S3). At the phylum level, the dominant bacterial phyla in the soil include Actinobacteria, Proteobacteria, Firmicutes, Chloroflexi, and Acidobacteria, with their relative abundances ranging from 83.23% to 88.09%. From Figure 14, it can be seen that there are some significant changes in the bacterial community composition in the S2 and S3 samples. In soils treated with TP-nZVI/BC and BH_4_-nZVI/BC, the relative abundance of Actinobacteria and Proteobacteria increased, especially in S2 (TP-nZVI/BC-treated), indicating that TP-nZVI/BC-treated soils effectively promoted the growth of certain heavy metal detoxifying and resistant bacteria, particularly Actinobacteria and Proteobacteria, which are known for their high resistance to heavy metals in contaminated environments. Actinobacteria is negatively correlated with heavy metal concentration and typically dominates in heavy metal-polluted environments, serving as an important organic matter degrader. Proteobacteria, on the other hand, contains various heavy metal detoxification genes, enabling them to participate effectively in heavy metal sensing and transformation. The increased abundance of these bacteria in S2 further demonstrates the positive impact of TP-nZVI/BC treatment on the microbial community [56,57]. Tea polyphenol modification significantly enhanced the remediation effect of TP-nZVI/BC. As a natural antioxidant, tea polyphenols can slow the formation of iron oxides and reduce oxidative damage to microorganisms, thereby aiding in the recovery and stability of the microbial community. In S2, tea polyphenol-modified BH_4_-nZVI/BC better maintained microbial diversity and functionality, especially promoting the growth of microbial communities with heavy metal detoxification capabilities. The antioxidant properties of tea polyphenols enabled TP-nZVI/BC to reduce the accumulation of oxidative byproducts during the remediation process, avoiding the negative effects of iron oxides on microorganisms. Therefore, tea polyphenol-modified BH_4_-nZVI/BC showed clear advantages in restoring soil health and improving heavy metal remediation efficiency. In contrast, the relative abundance of Firmicutes decreased in both S2 and S3 samples, suggesting that TP-nZVI/BC and BH_4_-nZVI/BC treatments may inhibit the growth of Firmicutes to some extent. Firmicutes are known for their strong resistance to heavy metals, especially Cd and As. Tea polyphenol-modified BH_4_-nZVI/BC could enhance the growth of certain heavy metal-resistant microorganisms by improving the soil environment while avoiding excessive proliferation of Firmicutes. Overall, the analysis in Figure 3 indicates that TP-nZVI/BC (S2) and BH_4_-nZVI/BC (S3) treatments can effectively alter the bacterial community composition in soil, promoting the growth of microbial groups with heavy metal detoxification functions. Particularly, tea polyphenol-modified BH_4_-nZVI/BC (S2) exhibited superior remediation effects, improving microbial community stability and enhancing soil heavy metal remediation capacity, highlighting the significant advantages of tea polyphenol modification in soil remediation.

### 3.9. Mechanism Analysis

It is difficult to separate a powder adsorbent after mixing with soil, making it difficult to analyze the adsorption mechanism of passivator on Cd in soil. To systematically explore the removal mechanism of Cd, a soil solution was used as the reaction system, and the solid residue obtained after immobilization was collected and measured by conducting XRD, FTIR, and SEM analysis.

**Figure 15:**
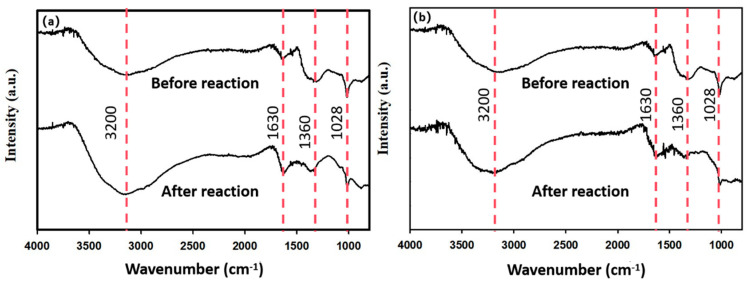
FTIR spectra of (**a**) BH4-nZVI/BC and (**b**) TP-nZVI/BC after immobilization with Cd (The indicator line of Wavenumber).

The functional groups on the surface of BH_4_-nZVI/BC and TP-nZVI/BC before and after the reaction were analyzed using FTIR spectroscopy, as shown in Figure 15. The broad absorption peak near 3460 cm^−1^ corresponds to the stretching vibration of -OH, indicating the presence of oxygen-containing functional groups on the surface of the BH_4_-nZVI/BC composite. The peak around 1630 cm^−1^ is attributed to C=O stretching vibrations, while the characteristic peaks at 1360 cm^−1^ and 1028 cm^−1^ are associated with -COOH and C-O vibrations, respectively [58]. No new peaks were observed after the reaction, suggesting that no new functional groups were introduced. However, the intensity of the peak at 1360 cm^−1^ exhibited varying degrees of change, which may be attributed to the complexation between Cd and the functional groups on the biochar surface.

**Figure 16:**
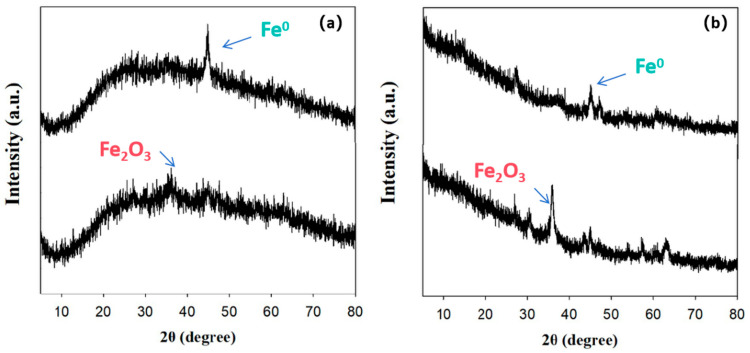
XRD pattern of (**a**) BH_4_-nZVI/BC and (**b**) TP-nZVI/BC before and after immobilization.

As shown in Figure 16, the XRD patterns of BH_4_-nZVI/BC and TP-nZVI/BC before the reaction reveal a distinct diffraction peak at 26.6°, corresponding to the (002) crystal plane of crystalline carbon. This peak is relatively broad, reflecting the structural characteristics of the biochar. Additionally, a characteristic diffraction peak of nanoscale zero-valent iron (nZVI) is observed at 44.9°, with a broad width, indicating the amorphous structure of the nZVI particles. After the reaction, the diffraction peak of nZVI becomes weaker and nearly disappears, while a new diffraction peak emerges at 35.63°. This change indicates a structural transformation of nZVI during the immobilization process. By comparing the XRD patterns with standard references, the new diffraction peak at 35.63° is likely attributed to the (110) crystal plane of Fe_2_O_3_ [59]. This suggests that nZVI undergoes a reaction during remediation, where Fe(II) is oxidized and released, leading to the formation of Fe_2_O_3_.

**Figure 17:**
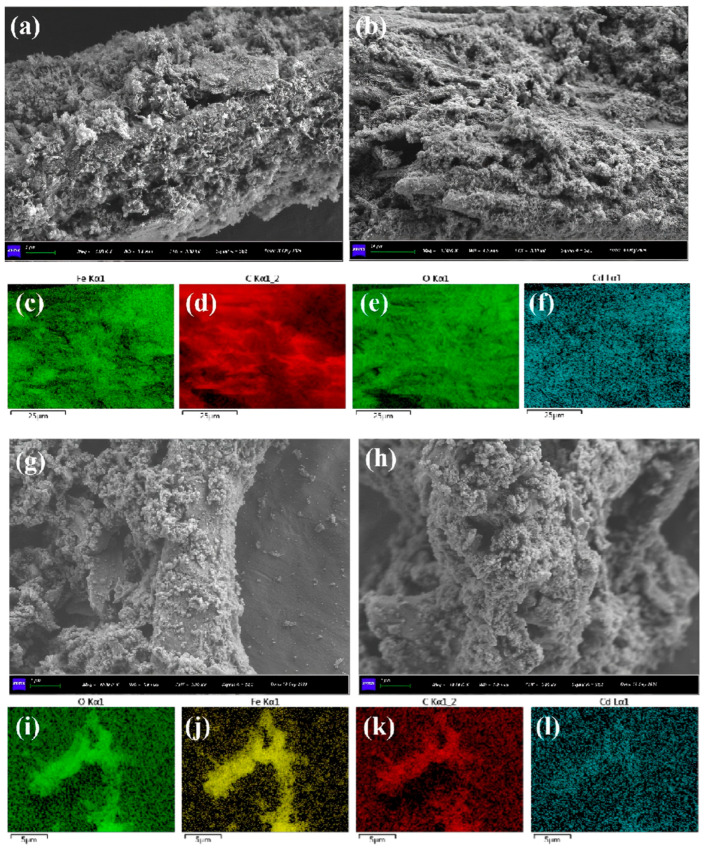
SEM images EDS mapping of BH_4_-nZVI/BC (**a**–**f**) and TP-nZVI/BC (**g**–**i**) after immobilization of Cd and (**j**–**l**) corresponds to the image features of (Fe, C, Cd) respectively.

The morphology of BH_4_-nZVI/BC after the reaction was analyzed using scanning electron microscopy (SEM) (Figure 17a–f). The observations revealed that the surface of BH_4_-nZVI/BC exhibited a compact structure after Cd immobilization, accompanied by significant aggregation of nZVI particles. Additionally, the porous structure of the original biochar was filled with nZVI particles. This transformation can be attributed to the formation of heavy metal complexes and iron oxides on the BH_4_-nZVI/BC surface during the reaction process. Energy-dispersive X-ray spectroscopy (EDS) mapping further demonstrated the uniform distribution of Cd on the BH_4_-nZVI/BC surface after adsorption, indicating a homogeneous distribution of other elements as well. The overlap between Cd and Fe elements suggests that a reaction occurred between zero-valent iron and Cd, such as a redox reaction. A similar phenomenon was also observed in TP-nZVI/BC (Figure 17g–l), where Cd was uniformly distributed on the material surface, further confirming the interaction between the immobilized Cd and the rXPSeactive components of the material.

**Figure 18:**
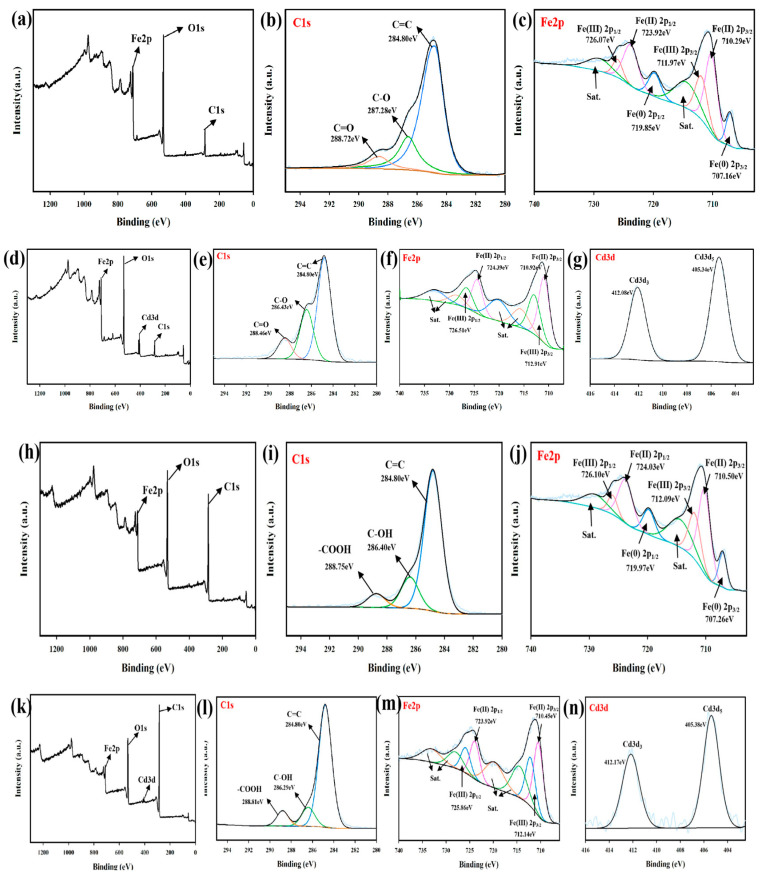
XPS spectra of BH_4_-nZVI/BC and TP-nZVI/BC before and after immobilization of Cd in the soil. For BH_4_-nZVI/BC, the spectra include (**a**) survey spectrum, (**b**) C 1s, and (**c**) Fe 2p before immobilization, and (**d**) survey spectrum, (**e**) C 1s, (**f**) Fe 2p, and (**g**) Cd 3d after immobilization. For TP-nZVI/BC, the spectra include (**h**) survey spectrum, (**i**) C 1s, and (**j**) Fe 2p before reaction, and (**k**) survey spectrum, (**l**) C 1s, (**m**) Fe 2p, and (**n**) Cd 3d after immobilization.

XPS was further used to analyze the BH_4_-nZVI/BC before and after the reaction (Figure 18). According to the full-scan XPS analysis, Cd3d peaks were detected in addition to Fe2p, O1s, and C1s peaks after the reaction, indicating the immobilization of Cd(II) on the surface of BH_4_-nZVI/BC. The high-resolution spectrum of C1s showed peaks at 284.80 eV, 287.28 eV, and 288.72 eV, corresponding to C–C, C–O, and C=O bonds, respectively. For Fe2p before the reaction, the peaks at 726.07 eV (Fe2p1/2) and 711.97 eV (Fe2p3/2) were assigned to Fe(III) in Fe_2_O_3_, while the peaks at 710.29 eV (Fe2p3/2) and 723.92 eV (Fe2p1/2) corresponded to Fe(II) in Fe_3_O_4_. The peaks at 707.16 eV and 719.85 eV were attributed to Fe(0), consistent with reported values, confirming the successful loading of nanoscale zero-valent iron on the surface of the BH_4_-nZVI/BC composite.

These results, in agreement with SEM, FTIR, and XRD characterizations, also indicated the presence of various iron oxides on the material surface, likely formed due to the oxidation of Fe(0) when exposed to air. This shell-core structure of zero-valent iron provides additional adsorption sites for contaminants. After the reaction, the Fe(0) peaks disappeared entirely, with all Fe(0) converted to Fe(II) and Fe(III), indicating that Fe(0) participated in the reaction between BH_4_-nZVI/BC and Cd(II). Similarly, XPS analysis was conducted for TP-nZVI/BC before and after the reaction. The full-scan spectrum showed Cd3d peaks after the reaction, confirming the immobilization of Cd(II) by TP-nZVI/BC.

Before the reaction, Fe2p peaks at 726.10 eV (Fe2p1/2) and 712.09 eV (Fe2p3/2) were attributed to Fe(III) in Fe_2_O_3_, while peaks at 710.50 eV (Fe2p3/2) and 724.03 eV (Fe2p1/2) were assigned to Fe(II) in Fe_3_O_4_. The peaks at 707.26 eV and 719.97 eV corresponded to Fe(0). After the reaction, the Fe(0) peaks disappeared completely, with all Fe(0) converted into Fe(II) and Fe(III), indicating that Fe(0) played a critical role in the immobilization of Cd(II). Additionally, the C1s spectra revealed abundant functional groups, such as C–C/C=C, C–O, and C=O, on the biochar surface before the reaction. These functional groups provided essential active sites for Cd adsorption and complexation. After the reaction, the intensities of the C–O and C=O signals increased, suggesting that Cd was likely bound to these functional groups via complexation. This demonstrates that biochar contributed significantly to the immobilization of Cd in the soil.

Through the above discussion, it can be concluded that the mechanisms of Cd immobilization in soil by BH_4_-nZVI/BC and TP-nZVI/BC are similar, primarily relying on the synergistic effects of nZVI’s strong reductive capacity and biochar’s adsorption and complexation capabilities. As the key active component, nZVI reacted Cd(II)^+^ to precipitates (e.g., Cd(OH)_2_ or Cd-containing iron oxides) via redox reactions, thereby significantly reducing Cd mobility and toxicity. XPS Fe2p spectra confirm the transformation of nZVI from zero-valent iron (Fe(0)) to oxidized forms (Fe(II)/Fe(III)), highlighting the role of nZVI as an electron donor during Cd reduction. Additionally, the disappearance of Fe(0) peaks in the XRD patterns further supports this observation. The iron oxide/hydroxide layer (e.g., FeOOH or Fe_2_O_3_) formed on the nZVI surface not only participates in co-precipitation with Cd but also stabilizes Cd through complexation. However, the activity of nZVI in soil may be limited due to particle aggregation and rapid oxidation. Biochar, as the support matrix for nZVI, provides critical physical and chemical support for Cd immobilization. Its abundant porous structure and high specific surface area offer stable sites for nZVI loading, reducing particle aggregation and maintaining high reaction activity. Additionally, biochar surfaces contain oxygen-rich functional groups (e.g., carboxyl, hydroxyl, and carbonyl groups), which bind with Cd(II) via electrostatic attraction or chemical complexation, further enhancing Cd immobilization [60]. The enhancement of these functional groups after the reaction, as indicated by the XPS C1s spectra, underscores their crucial role in the immobilization process (Equations (1)–(3))

(1) $${Biochar}_{surface}-{COOH}^{:}+{Cd}^{2+}\to Biochar-{COOCd}^{+}+2\cdot OH+H^{+}$$

(2) $$\equiv {FeO}^{-}+{Cd}^{2+}\to {\equiv FeOCd}^{+}+OH+H^{+}$$

(3) $$2OH+{Cd}^{2+}\to {Cd(OH)}_{2}$$

In addition, the buffering capacity of biochar may help regulate soil pH, creating favorable environmental conditions for Cd precipitation and adsorption reactions [61]. Simultaneously, TP-nZVI/BC benefits from the influence of tea polyphenols introduced during the preparation process, which enhances the material’s anti-aging properties and reduces the leaching of heavy metals during TCLP toxicity testing. Tea polyphenols, used as a reducing agent in the preparation of TP-nZVI/BC, offer significant advantages in enhancing the stability and remediation performance of the material.

To determine whether tea polyphenols are present on nZVI, we conducted FTIR on zero-valent iron prepared using tea polyphenols as a reducing agent. Based on the FTIR spectra shown in the Figure S2, a detailed analysis of the characteristic peaks of TP, nZVI, and TP-nZVI was performed. First, the spectrum of tea polyphenols shows a broad absorption peak around 3400 cm^−1^, corresponding to the stretching vibration of the hydroxyl group (-OH), indicating the presence of multiple phenolic hydroxyl groups in the tea polyphenols. These groups play a crucial role in antioxidant activity. The broadness of this peak also suggests that these hydroxyl groups may form hydrogen bonds with water or other molecules. Additionally, the absorption peak near 1500 cm^−1^ is associated with the stretching vibration of C=C in the aromatic ring, reflecting the structure of the aromatic rings in tea polyphenols, which are a key source of activity and are crucial for the reduction reaction with iron. The absorption peak at 1600 cm^−1^ corresponds to the stretching vibration of C=O, which may be related to the oxidized derivatives or acylation products of tea polyphenols. For nZVI, the IR spectrum shows a peak around 600 cm^−1^, which corresponds to the stretching vibration of the Fe-O bond, indicating the possible formation of iron oxides or iron hydroxides on the surface of nZVI during the preparation process. Moreover, the broad absorption peak around 3400 cm^−1^ is related to the stretching vibration of Fe-OH, indicating that nZVI surface may have adsorbed water or formed hydroxyl groups.

In the spectrum of TP-nZVI, the hydroxyl stretching vibration peak at 3400 cm^−1^ remains, indicating that the phenolic hydroxyl and hydroxyl (-OH) groups from tea polyphenols are still present, suggesting that the modification by tea polyphenols is retained in TP-nZVI. The antioxidant properties of tea polyphenols effectively prevent the oxidation of nZVI, and their phenolic hydroxyl and hydroxyl groups provide stability, reducing the formation of iron oxides and preventing their negative effects on microorganisms. Furthermore, the functional groups of tea polyphenols, particularly the phenolic hydroxyl and hydroxyl groups, are capable of forming coordination complexes with heavy metal ions like Cd(II), further reducing the mobility and bioavailability of heavy metals, thereby enhancing the immobilization effect. So, FTIR spectroscopy analysis revealed that the surface of TP-nZVI/BC contains characteristic functional groups of tea polyphenols, such as -OH, C=O, and phenolic hydroxyl groups. These functional groups not only effectively reduce Fe(III) to Fe(0) and help stabilize the nZVI particles but also play an important role in the immobilization of Cd.

**Figure 19:**
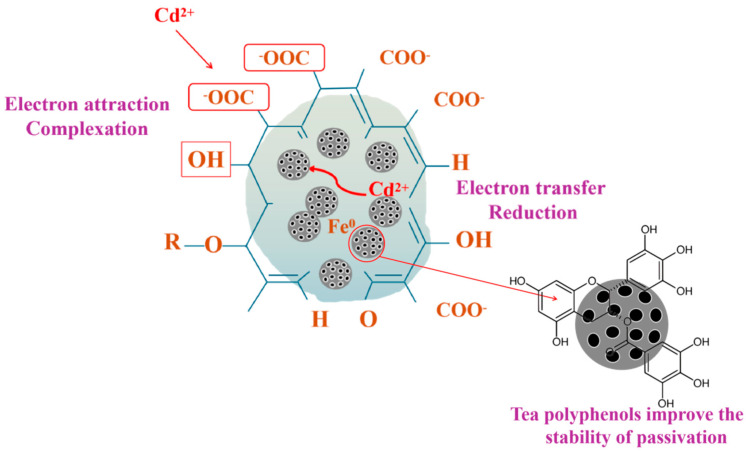
Immobilization mechanism of Cd onto BH_4_-nZVI/BC and TP-nZVI/BC.

Specifically, functional groups such as hydroxyl and phenolic hydroxyl have strong complexation abilities and can form coordination complexes with Cd(II), further reducing the bioavailability and mobility of Cd [62]. Additionally, the antioxidant properties of tea polyphenols reduce the formation of iron oxides, preventing their negative impact on soil microorganisms and helping to maintain microbial diversity [63]. Therefore, the modification by tea polyphenols not only enhances the remediation effectiveness of TP-nZVI/BC but also strengthens its stability through the rich functional groups, particularly hydroxyl and phenolic hydroxyl groups, significantly extending the durability of Cd immobilization and enhancing its long-term stability and ecological compatibility in environmental remediation (The mechanism is illustrated in Figure 19).

## 4. Conclusions

BH_4_-nZVI/BC prepared using NaBH_4_ and TP-nZVI/BC both proved effective for the remediation of Cd(II)-contaminated soil. Cd was immobilized through the strong reductive capacity of nZVI, transforming it into less toxic and less mobile forms, while the hydroxyl, carboxyl, and phenolic groups of biochar further enhanced immobilization via adsorption and complexation. Between the two materials, BH_4_-nZVI/BC exhibited greater short-term immobilization efficiency, whereas TP-nZVI/BC demonstrated stronger long-term stability and ecological compatibility. Tea polyphenols, acting as both reducing agents and stabilizers, not only facilitated the synthesis of nZVI but also prevented nanoparticle agglomeration and oxidation, preserved Fe^0^ activity, and promoted microbial recovery, thereby ensuring more sustainable remediation. Taken together, these results highlight that although BH_4_-nZVI/BC can deliver rapid immobilization, TP-nZVI/BC represents the more favorable option for practical and long-term soil remediation applications.

## Figures and Tables

**Table 1:** Soil microbial diversity index.

Sample ID	Richness	Chao1	ACE	Shannon	Simpson	Good’s Coverage
S0	942	1011.76	1000.06	4.88	0.96	0.9958
S1	795	891.87	849.81	5.34	0.99	0.9963
S2	776	853.17	819.94	4.88	0.98	0.9976
S3	706	768.34	749.52	3.32	0.87	0.9983

## Supplementary Materials

The following supporting information can be downloaded at: https://www.mdpi.com/article/10.3390/nano15191460/s1, Figure S1: Flowchart of Cd immobilization and analysis procedures; Figure S2: FTIR of Tea Polyphenols, nZVI and TP-nZVI.
