# Supplementary material for: Green Synthesis of Biochar-Supported Nanoscale Zero-Valent Iron Using Tea Polyphenol for Efficient Cadmium Immobilization in Soil
Source: Nanomaterials (Basel). 2025 Sep 23;15(19):1460. doi: 10.3390/nano15191460 (PMC12525840; doi:10.3390/nano15191460)
Supplement: Supplementary file 1 [file nanomaterials-15-01460-s001.zip › nanomaterials-3814143-supplementary.pdf]

Green Synthesis of Biochar-Supported Nanoscale Zero-Valent Iron Using Tea Polyphenol for Efficient Cadmium Immobilization in Soil

Ziyong Jia [1,2], Huizi Wang [1,2], Shupei Yuan [1,2], Weifeng Zhang [1,2] and Daijun Zhang [1,2,*]

[1] State Key Laboratory of Coal Mine Disaster Dynamics and Control, Chongqing University, Chongqing 400044, China; jiaziyong1121@163.com (Z.J.); 202317131192@stu.cqu.edu.cn (H.W.); 202317021030@stu.cqu.edu.cn (S.Y.); 20211701030@cqu.edu.cn (W.Z.)

[2] Department of Environmental Science, College of Environment and Ecology, Chongqing University, Chongqing 400030, China

* Correspondence: dzhang@cqu.edu.cn

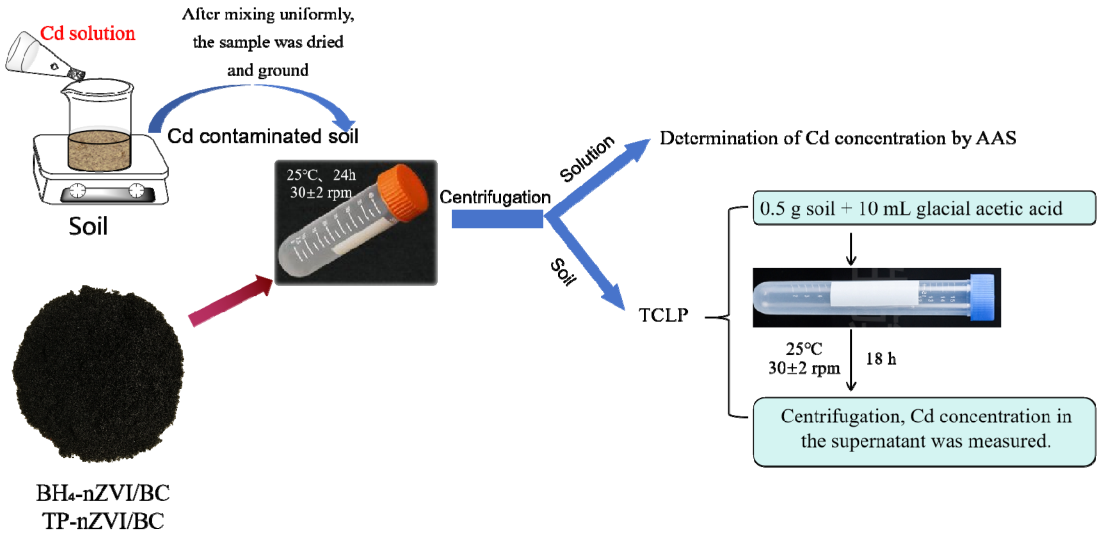

Figure S1 Flowchart of Cd immobilization and analysis procedures

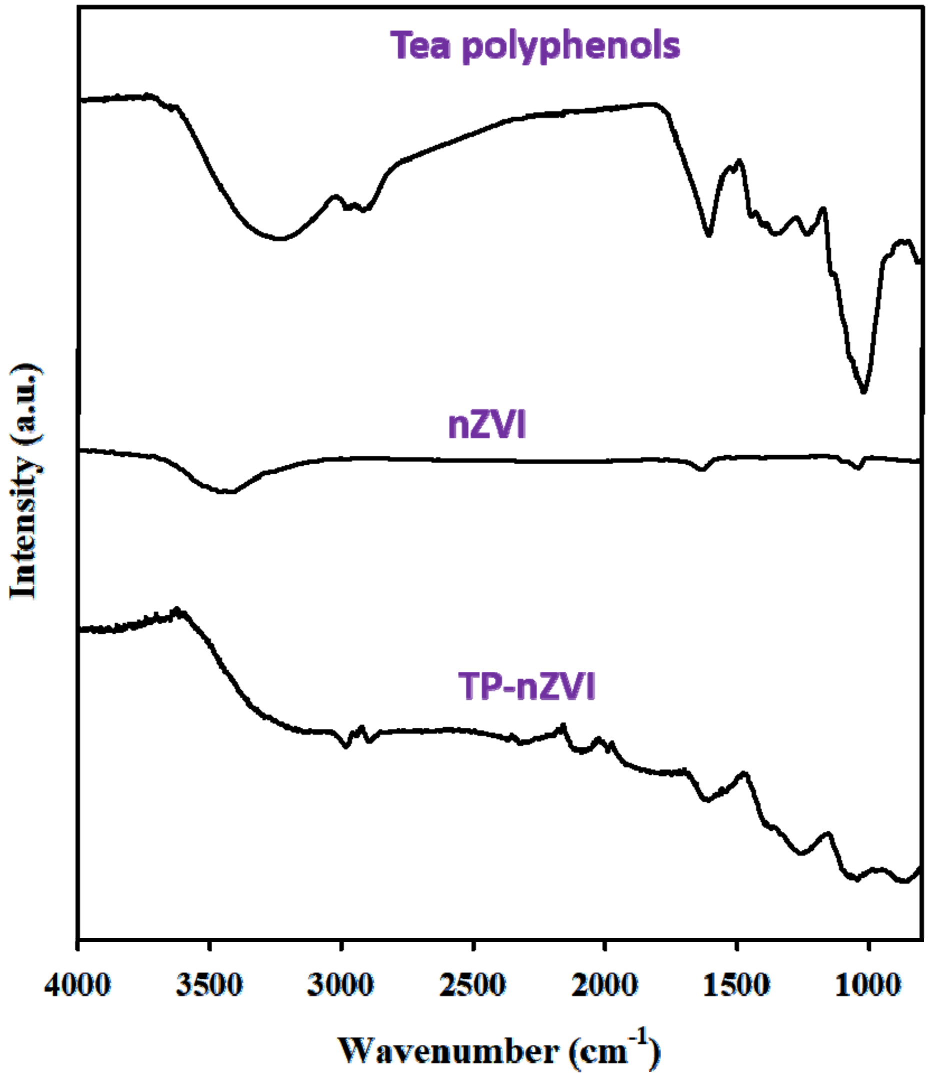

Figure S2 FTIR of Tea Polyphenols, nZVI and TP-nZVI.
